# Habituation and novelty detection fNIRS brain responses in 5‐ and 8‐month‐old infants: The Gambia and UK

**DOI:** 10.1111/desc.12817

**Published:** 2019-03-13

**Authors:** Sarah Lloyd‐Fox, Anna Blasi, Samantha McCann, Maria Rozhko, Laura Katus, Luke Mason, Topun Austin, Sophie E. Moore, Clare E. Elwell, The BRIGHT project team

**Affiliations:** ^1^ Centre for Brain and Cognitive Development Birkbeck University of London London UK; ^2^ Department of Psychology University of Cambridge Cambridge UK; ^3^ Department of Medical Physics and Biomedical Engineering University College London London UK; ^4^ Medical Research Council The Gambia at the London School of Hygiene and Tropical Medicine London UK; ^5^ Great Ormond Street Institute of Child Health University College London London UK; ^6^ Department of Neonatology The Rosie Hospital, Cambridge University Hospitals NHS Foundation Trust Cambridge UK; ^7^ Department of Women and Children’s Health Kings College London London UK

## Abstract

The first 1,000 days of life are a critical window of vulnerability to exposure to socioeconomic and health challenges (i.e. poverty/undernutrition). The Brain Imaging for Global Health (BRIGHT) project has been established to deliver longitudinal measures of brain development from 0 to 24 months in UK and Gambian infants and to assess the impact of early adversity. Here results from the Habituation‐Novelty Detection (HaND) functional near‐infrared spectroscopy (fNIRS) task at 5 and 8 months are presented (*N* = 62 UK; *N* = 115 Gambia). In the UK cohort distinct patterns of habituation and recovery of response to novelty are seen, becoming more robust from 5 to 8 months of age. In The Gambia, an attenuated habituation response is evident: a larger number of trials are required before the response sufficiently suppresses relative to the response during the first presented trials. Furthermore, recovery of response to novelty is not evident at 5 or 8 months of age. As this longitudinal study continues in The Gambia, the parallel collection of socioeconomic, caregiving, health and nutrition data will allow us to stratify how individual trajectories of habituation and recovery of response to novelty associate with different risk factors and adaptive mechanisms in greater depth. Given the increasing interest in the use of neuroimaging methods within global neurocognitive developmental studies, this study provides a novel cross‐culturally appropriate paradigm for the study of brain responses associated with attention and learning mechanisms across early development.

**RESEARCH HIGHLIGHTS**

- Global health research has been limited to the use of behavioural assessments to measure the effect of exposure to early adversity (socioeconomic and health challenges)
- Functional near‐infrared spectroscopy (fNIRS) is a portable, non‐invasive, low‐cost imaging technique that provides a new avenue for measuring brain function in low‐resource settings.
- A novel cross‐culturally appropriate paradigm demonstrated habituation and novelty detection brain responses in 5‐ and 8‐month‐old infants in the UK.
- In the Gambia, habituation brain responses were attenuated, and a recovery of response to novelty absent, at the group level, at 5–8 months of age.

## 1 INTRODUCTION

The United Nations Sustainable Development Goals have identified the reduction of poor cognitive development during childhood in low‐ and middle‐income countries (LMICs) as a key priority for global health research and interventions (UN, 2015). Infants in resource‐poor settings may frequently be exposed to a range of social, environmental, nutritional, and health challenges. According to a recent study, one third of children in developing countries fail to reach their developmental milestones in cognitive and/or socioemotional growth, with the largest number of affected children in sub‐Saharan Africa (McCoy et al., 2016) This means that over 80 million children in LMICs fail to develop a core set of age‐appropriate skills that allow them to maintain attention, understand and follow simple directions, communicate and cooperate with others, control aggression, and solve complex problems. Compromised development of these skills may have a significant impact on their subsequent academic achievement, mental health and economic status, and consequently their potential to lead full and productive lives and support future generations.

The first 1,000 days of life (from conception to 2 years of age) represents a critical window for brain and nervous system development in humans and have been described “as a foundation and catalyst of human development in the balance of the life course” (Bornstein, 2014). While only a fraction of our lifespan, it is characterized by prodigious physiological, psychological and physical change. From the first moments of life, the as‐yet physically immature neonate manifests stimulus specific physiological, behavioural and cortical responses across the senses: vision (Farroni et al., 2013; Farroni, Csibra, Simion, & Johnson, 2002), audition (DeCasper & Fifer, 1980; Ockleford, Vince, Layton, & Reader, 1988), smell (Bartocci et al., 2000) and touch (Lejeune et al., 2010). In the weeks and months that follow, infants assimilate information through multiple interactions with their world; developing and honing physical, expressive and receptive skills. The brain is at its most plastic during this time, and these changes are the outward manifestation of increasing cortical specialization to its particular environment (Johnson, 2011). Over the course of an infant's individual development the brain adapts both to the general features of the environment shared by others, and to the individual circumstances into which it is born (Johnson, Jones, & Gliga, 2015).

How the individual brain adapts during a lifespan in relation to early environmental adversity, can be thought of in different ways. First, as related to “resilience,” or rather the ability of the individual to avoid deviation from a typical developmental trajectory in the face of environmental challenges (Karatsoreos & McEwen, 2013). Second as related to “ontogenic adaptation” whereby an individual develops in such a way that allow an optimal fit between environmental demands and brain functioning. Rather than making the assumption that one trajectory is favourable over the other, this notion allows for adaptive advantages to be experienced in different contexts (Johnson et al., 2015).

This can be conceptualized along different time scales. Adaptability of the brain may be essential for normal function and brain development in response to events ranging from the acute (e.g. premature birth), to the medium‐term (e.g. response to acute periods of infection leading to undernutrition/sleep disruption at one age point) to the chronic (e.g. continued presence of early adversity such as undernutrition or poverty throughout childhood). Furthermore, these factors may work synergistically whereby an infant born prematurely may be at greater risk of infectious illnesses and may consequently experience prolonged undernutrition (Nelson, 2017). In addition factors such as poverty can be extrinsically bound to further psychosocial risk factors (Jensen, Berens, & Nelson, 2017), such as child maltreatment, domestic violence or stress (Giovanelli, Reynolds, Mondi, & Ou, 2016). With perhaps more widespread consequences, poverty will also require caregivers to allot an increased allocation of time to income generation and household duties and thus decreased opportunities for infant‐caregiver interactions and decreased access to family resources (Milteer, Ginsburg, Council On Communications And Media, & Committee On Psychosocial Aspects Of Child And Family Health, 2012; Worku et al., 2018).

While many studies suggest that the presence of these risk factors in infancy has a lasting impact throughout the life course (Hackman & Farah, 2009; Martorell et al., 2010; Victora et al., 2008), very little is known about the neural bases of these early deficits, particularly in low‐ and middle‐income countries. This may be partly a consequence of limited availability of child‐friendly portable brain imaging methods (Isaacs, 2013; Lloyd‐Fox et al., 2017). Therefore the investigation of the developing brain in remote rural or urban “field based” settings has until recently been broadly limited to behavioural assessments (Georgieff, 2007; Sabanathan, Wills, & Gladstone, 2015). Importantly, while behavioural responses can be measured from birth (i.e. visual attention, sucking modulation) early in development these responses may be more variable or subtle and therefore less reliable than a neural measure. Furthermore, observable changes in an individual's behaviour may not be measurable in pre‐verbal infants and/or become predictive of developmental outcome until much later in development (i.e. toddlerhood)—despite significantly earlier changes in brain functional and anatomical specialization—thereby missing a window of vulnerability for initiating an intervention. Neural measures may allow us to access this window and provide earlier diagnostic tools.

To contribute to our understanding, it is therefore imperative neurocognitive development is studied longitudinally and from an early age, taking contemporaneous measurements of brain function in parallel with measurements of exposure to environmental challenges. The alternative—assessing impairment at school age and attempting to retrospectively decode the cascading effects of early environmental insults—is unlikely to reveal clear and reliable targets for intervention.

The Gambia, the smallest country on the African continent, has, for over 70 years, partnered with the UK Medical Research Council (MRC) to conduct research to further understand the impact of undernutrition and poverty and provide interventions to promote healthy growth in the population. This research focus is partly due to the contrasting weather patterns which alternate between 6 months where rain occurs and six months of extreme dryness, which directly affects the availability of key nutrients (Moore et al., 1997). The majority (60%) of the roughly 2 million inhabitants live in the coastal regions surrounding the capital, whereas the remainder of the population live rurally, often supporting themselves through subsistence farming (Hennig et al., 2017). The Gambia is one of the lowest ranking countries with regard to gross national income, years of schooling and life expectancy, with over half of adults in The Gambia never having received formal education (Hennig et al., 2017). For the last decade, education level has risen as there is now free universal education which 97% of children attend to primary level (The Gambia Government National Education Statistics by Gender, 2016). Marriages are commonly polygamous with over half of wives living with one or two co‐wives (Hennig et al., 2017). Over the past decades infant and child mortality has decreased, birth spacing has increased, and overall family size has gone down (Nabwera, Fulford, Moore, & Prentice, 2017).

This research was conducted at the Medical Research Council Gambia Unit at the London School of Hygiene and Tropical Medicine (MRCG at LSHTM) in the Gambia (www.mrc.gm; www.ing.mrc.ac.uk). One of their rural research sites is located in the West Kiang District, which is 145 km inland from the capital of The Gambia. The field station where the study took place is reached by 4 × 4 vehicles on unmade roads and located within a remote inland village, Keneba. Due to its isolation the field station in the village maintains all facilities necessary for research and clinical care (generator powered electricity, water supplied by bore hole, satellite communication). The community in this region relies predominantly on subsistence farming and, thus, eating patterns and income vary greatly between the annual wet and dry seasons (Hennig et al., 2017; van der Merwe et al., 2013). Additionally, there is a high prevalence of infectious disease (van der Merwe et al., 2013). The combination of prenatal growth retardation, poor‐quality and often contaminated foods and high levels of infection cause moderate to severe growth faltering (failure to thrive) in height and weight gain from around three months of age in the local population of West Kiang (Lunn, 2000; Lunn, Northrop‐Clewes, & Downes, 1991; van der Merwe et al., 2013). Growth faltering is most severe beyond the first six‐months of life, when non‐breast milk feeds are introduced (Eriksen et al., 2017).

Over the last 6 years the use of infant friendly neuroimaging techniques has been pioneered at this MRCG at LSHTM field station in The Gambia (Begus et al., 2016; Lloyd‐Fox et al., 2017, 2014; Papademetriou et al., 2014). These proof‐of‐concept studies have led to the establishment of a prospective longitudinal study to measure early neurocognitive development during the first 2 years of life at two parallel sites, in the UK and The Gambia (see Supplementary information Data S1 for demographical information on The Gambia). This project aims to implement brain imaging measures (functional Near‐InfraRed Spectroscopy [fNIRS] and electroencephalography [EEG]), population‐specific neurocognitive developmental measures (i.e. Mullen Scales of Early Learning [MSEL] and the Communicative Development Inventory [CDI]) and family‐caregiving assessments (caregiver‐infant interaction videos and questionnaires) into a framework of regular collection of biological, socioeconomic, parental health and nutritional data at both sites. The data presented in this study are from the full UK cohort (*N* = 62) and the first 115 infants studied in The Gambia^1^ and longitudinal data collection is ongoing. The longitudinal design includes nine data collection phases; antenatal (32–36 weeks gestation), and postnatal—1–3, 7–14 days, 1, 5, 8, 12, 18 and 24 months of age. In the full‐scale BRIGHT study, the cohort size in The Gambia was selected—to provide adequate power to support within‐cohort comparisons—on the assumption that approximately 25%–30% of the cohort will be stunted (z‐score of length‐for‐age <2 standard deviations below the WHO reference) by 2 years of age (Nabwera et al., 2017). While a second cohort within The Gambia (i.e. infants from an urban coastal where undernutrition is less prevalent) was originally considered, there are considerably varying environmental and cultural factors to take into account (which are more rapidly changing relative to rural areas due to economic development, and the associated health and nutrition transition). Therefore a larger cohort was purposefully selected within the rural district. The purpose of the BRIGHT study is not to draw comparisons between the UK and Gambia cohorts. This design will firstly allow the modelling of longitudinal changes in brain function, cognitive development and growth within the rural Gambian population. Second, through the collection of parallel behavioural and environmental data it will allow the identification of critical developmental moderators, mediators and markers of resilience. Third, given that neuroimaging data provide the backbone of this study, it was essential that a UK cohort was established to measure the longitudinal developmental trajectories of the different fNIRS and EEG paradigms (some of which have not been studied at these ages before). This was chosen to broadly match the context of previously acquired developmental neuroimaging data, given that to date the vast majority of research of this kind has been undertaken in Western, Educated, Industrialised, Rich and Democratic (WEIRD) populations (Henrich, Heine, & Norenzayan, 2010).

fNIRS data are acquired at each visit from 1 to 24 months of age (6 time‐points): the design includes measures of visual and auditory social selectivity, selective attention through measures of habituation and recovery of response to novelty, working memory development and functional connectivity. This broad range of assessments will enable typical pathways of brain development to be mapped across a range of cognitive domains within this rural Gambian population. Secondarily, this project will investigate whether key environmental factors (i.e. undernutrition, poverty, parent–infant interaction) have an adverse or positive impact on brain development within this cohort across multiple cognitive functions or whether there are crucial periods of sensitivity for particular sub‐domains of early processing and specialization.

Over the last 20 years research has shifted focus to try and understand whether poverty associated risk factors are associated with differences in specific neurocognitive systems such as memory, language or attention (Farah et al., 2006; Mezzacappa, 2004; Noble, Norman, & Farah, 2005) While largely restricted to the study of low SES within high‐income countries, this behavioural research with school‐aged children has led authors to propose that early development of these foundational skills may have cascading effects on later development. Motivated by evidence of behavioural deficits in these neurocognitive domains, several recent EEG studies have reported evidence of differences in neural mechanisms of attention in children (D'Angiulli, Herdman, Stapells, & Hertzman, 2008; Stevens, Lauinger, & Neville, 2009) and younger infants (Tomalski et al., 2013) living within low SES settings.

This study implements a Habituation and Novelty detection (HaND) paradigm at 1 to 24 months of age to allow the investigation of the development of discriminatory neural responses associated with—attention, learning and memory mechanisms—across infancy and early toddlerhood. Here, data are presented from two of the longitudinal time points (5 and 8 months) across both research sites (the UK and The Gambia). The HaND paradigm is designed to allow the measurement of habituation (or repetition suppression) responses, and recovery of response and subsequent novelty detection to a change in stimuli. Such effects have been observed in single cell recordings and adult neuroimaging studies (Desimone & Duncan, 1995; Grill‐Spector, Henson, & Martin, 2006). In adult studies neural repetition suppression has been associated with cognitive processes of discrimination or learning, however, this is less well understood in infancy. Following on from earlier behavioural work (DeCasper & Fifer, 1980; DeCasper & Spence, 1986; Mehler et al., 1988), which evidenced newborns’ ability to detect a change in speaker during passive listening to spoken sentences, several recent fNIRS studies have shown that repeated exposure to identical stimuli, either visual or auditory, produces neural habituation in very young infants (Benavides‐Varela et al., 2011; Bouchon, Nazzi, & Gervain, 2015; Nakano, Watanabe, Homae, & Taga, 2009), and a recovery of response to novelty (e.g. a change in speaker) (Benavides‐Varela et al., 2011; Nakano et al., 2009). For this study, a novel fNIRS paradigm was developed, designed to be objective and appropriate for use across different populations, to extend the applicability to older, awake infants and enable the investigation of developmental trajectories of habituation (repetition suppression) and novelty detection. All these previous fNIRS studies on habituation have been undertaken with newborns (one study at 3 months of age), while they are sleeping, under laboratory conditions. In this study infants were awake, and this paradigm was presented as part of a battery of other fNIRS tasks. Therefore the design of previous studies (Benavides‐Varela et al., 2011; Nakano et al., 2009) was adapted with the intention of (a) increasing data retention, (b) utilizing a situation more similar to everyday learning situations (Benavides‐Varela et al., 2017), (c) creating a paradigm that would be more appropriate for application in a noisier and variable environment such as that often encountered in low‐resource global health research contexts (Sabanathan et al., 2015) and (c) allowing the investigation of responses in participants across a broad age range from 0 to 24 months of age. The paradigm employed a naturalistically presented and engaging sentence, spoken by a culturally appropriate adult (i.e. UK or Gambian), within an environment containing naturalistic engaging (and potentially interfering) additional visual stimuli. Infants listened to a repeating spoken sentence during familiarization trials, which switched to a novel speaker during novelty trials, then returned to the familiar speaker at the end of the session. The rationale for using a change in voice was that infants in both cultures should be capable of discriminating pitch changes induced by a change in gender of the speaker; these are salient features of the infants’ environment. This rapid repetition suppression (habituation) model was adopted rather than a paradigm with a delay between adaptor and target as it has been suggested that these are less demanding in terms of memory capacity and may be more sensitive to developmental changes within infant populations (Nordt, Hoehl, & Weigelt, 2016). Furthermore, the feasibility of using sentence‐level adaptation neuroimaging paradigms has been previously shown in adults (Dehaene‐Lambertz et al., 2006), and differential haemodynamic responses to a change in speaker from male/female has been evidenced from very early in life in infants born preterm at 28–32 weeks gestation (Mahmoudzadeh et al., 2013). The fNIRS arrays were designed so that responses could be recorded over auditory associative brain regions including the inferior frontal gyrus (IFG), middle and superior temporal regions and extending to the temporo‐parietal junction across both cohorts (Lloyd‐Fox et al., 2014). These regions have been previously linked with auditory repetition suppression and novelty detection in early infancy (Benavides‐Varela et al., 2011; Bouchon et al., 2015; Mahmoudzadeh et al., 2013; Nakano et al., 2009) and adulthood (Belin & Zatorre, 2003; Dehaene‐Lambertz et al., 2006), and subsequent recovery of response to novel speech (Benavides‐Varela et al., 2011, 2017; Bouchon et al., 2015; Dehaene‐Lambertz et al., 2006).

In this study, infants were tested at 5 and 8 months of age at two sites (The UK and The Gambia). It was hypothesized that initial responses would manifest over auditory associative regions, diminish following repetition and recover as the novel stimulus was detected. Furthermore, it was hypothesized that increasingly rapid habituation (and therefore more mature and specialized neural processing) would be evident as the infants become older. An increasing number of studies are reporting adverse effects of poverty associated risk factors on the developing brain (D'Angiulli et al., 2008; Stevens et al., 2009; Tomalski et al., 2013; Xie et al., 2018). While current analyses cannot assess the direct relation to these risk factors as data collection is not complete for longitudinal growth and risk modelling, the diverse exposure to environmental challenges (i.e. poverty, undernutrition) present in the Gambian cohort allowed the investigation of these developmental brain responses within a novel rural low‐resource population in which it was hypothesized neural discriminatory responses could be affected.

## 2 METHODS

### 2.1 Participants

Participants were recruited from two sites: Cohort 1 in the UK and Cohort 2 in The Gambia and tested at 5 and 8 months of age, between June 2016 and February 2018.

For Cohort 1 families were recruited during pregnancy at their antenatal visit to the Rosie Hospital, Cambridge University Hospitals NHS Foundation Trust at 32–36 weeks’ gestation. Once per week during the recruitment phase all families visiting for their antenatal visit with a healthy pregnancy were approached and given information about the study. Interested families were followed up via email/phone and recruited into the study. In total, 61 families were recruited into Cohort 1 of this study. The majority of families lived either in the university town or in surrounding urban or rural communities within a 20‐mile radius. The study was approved by the National Research Ethics Service Committee East of England (REC reference 13/EE/0200), and informed written consent was obtained from parents of infants to participate.

For Cohort 2 families were recruited during pregnancy from the village of Keneba and neighbouring villages in the rural West Kiang District in The Gambia. Families were identified using the West Kiang Demographic Surveillance System (Hennig et al., 2017). Ethical approval was given by the joint Gambia Government—MRCG Ethics Committee, and informed consent was obtained in writing, or via thumbprint if individuals were unable to write, from all parents/carers prior to participation.

To be included in the present fNIRS study, infants from both cohorts must have been born at term (37–42 weeks’ gestation) and within the UK at normal birth weight (>2.5kg). In the West Kiang District in The Gambia, a combination of prenatal growth retardation, poor‐quality and often contaminated foods and high levels of infection cause moderate to severe growth faltering in height and weight gain from around 3 months of age in the local population (Lunn et al., 1991; Lunn, 2000; van der Merwe et al., 2013). While infants with an indication of severe growth faltering (weight‐for‐height (WHZ) or head circumference (HCZ) z‐score less than −3 according to WHO standards) were not excluded, growth measures were recorded at each time point (see Table 1). Overall, four infants had z‐scores falling below −3 in WHZ or HCZ, of which only one had scores below −3 at more than one age point. Furthermore, while there are multiple ethnic groups in the country, mostly associated with their own language and cultural customs, the largest proportion of the population belongs to the Mandinka ethnic group (Jukes & Grigorenko, 2010). To avoid confounds in the sample due to translation of stimuli and questionnaires into multiple languages, it was decided to only enrol families of the Mandinka group into this study.

**Table 1:** Summary of participant characteristics for the UK and Gambian Cohorts

	UK cohort			GM cohort		
Characteristics	1 month	5 months	8 months	1 month	5 months	8 months
Sex (m/f)	22/20	21/18	21/22	30/43	40/41	30/30
Age (days)	33.29 ± 6.33	155.79 ± 7.51	250.91 ± 9.34	36.44 ± 6.08	160.25 ± 19.61	245.20 ± 10.75
Weight (kg)	4.30 ± 0.53	7.15 ± 0.85	8.38 ± 1.09	4.23 ± 0.52	6.75 ± 0.79	7.59 ± 0.88
Length (cm)	53.76 ± 2.09	64.02 ± 4.02	69.35 ± 2.77	53.04 ± 2.77	64.14 ± 2.08	68.14 ± 3.64
Head circumference (H‐C, cm)	37.77 ± 1.03	42.96 ± 0.93	45.00 ± 1.25	36.64 ± 1.01	41.24 ± 1.19	43.18 ± 1.31
MUAC (cm)	11.70 ± 1.18	14.37 ± 1.19	15.04 ± 1.37	11.61 ± 0.91	13.25 ± 1.11	13.42 ± 1.04
Growth anthropometric z‐scores^a^						
Weight‐for‐age (WAZ)	N/A	N/A	N/A	−0.55 ± 0.93	−0.64 ± 93	−0.87 ± 0.96
Length‐for‐age (HAZ)	N/A	N/A	N/A	−0.93 ± 1.39	−0.45 ± 0.92	−0.79 ± 1.56
H‐C‐for‐age (HCZ)	N/A	N/A	N/A	−0.52 ± 0.84	−0.67 ± 0.84	−0.65 ± 0.94
Weight‐for‐length (WHZ)	N/A	N/A	N/A	0.28 ± 1.11	−0.41 ± 1.02	−0.59 ± 1.22

Note

MUAC: mid upper arm circumference.

a With the use of WHO reference curves.

**Figure 1:**
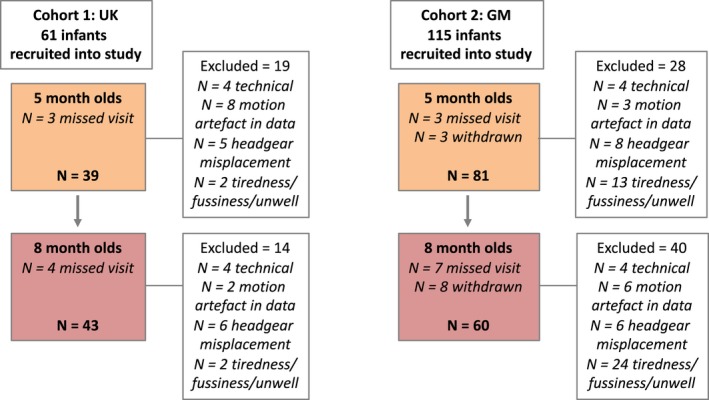
A flowchart illustrating the sample sizes of each Cohort (UK and The Gambia), those tested at each age point, and reasons for exclusion/withdrawal

This study presents Habituation‐Novelty detection (HaND) data from the first two postnatal longitudinal time points (5 and 8 months) to be conducted while the infants were awake (as at 1 months they were testing while asleep). Following each session, infant's data could be excluded for the following reasons; due to (a) motion artefact in the data, (b) fussiness or illness resulting in the session not running or finishing early, (c) technical—experiment error or piloting phase, (d) improper placement or slippage of headgear, (e) missed visit or (f) family withdrew from study (see Figure 1). Therefore for *Cohort 1 (UK),* 62 infants were recruited in the study (this is the full Cohort for the UK BRIGHT study). Of the 62 infants initially recruited (see Figure 1) 58 were tested at 5‐months with 39 contributing valid data sets (67.2% valid: 19 female, mean age = 155.79 days, *SD* = 7.51); and 57 were tested at 8‐months with 43 contributing valid data sets (75.4% valid: 22 female, mean age = 250.91 days, *SD* = 9.34)—see Table 1.

For *Cohort 2 (Gambia),* 115 infants were recruited in the study (this is a subcohort of the full 225 infants recruited in the Gambia for the BRIGHT study who had reached 8 months and for whom data were collected and analysed). Of the 115 infants initially recruited (see Figure 1) 109 were tested at 5 months with 81 contributing valid data sets (74.3% valid: 41 female, mean age = 160.3 days, *SD* = 10.4); and 100 were tested at 8 months with 60 contributing valid data sets (60% valid: 29 female, mean age = 245.2 days, *SD* = 10.75)—see Table 1. Note that in the Gambia, as the infants became older a higher number fussed out before the study began or very soon after the beginning of the study, and several of these infants were referred to the clinic as they were unwell. Therefore this exclusion criterion was higher than in the UK.

### 2.2 Experimental procedures

**Figure 2:**
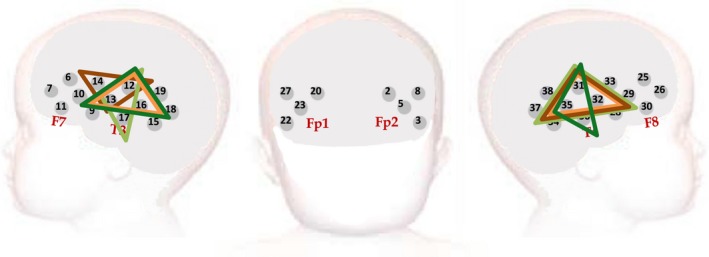
Representation of the headgear placement on a schematic of the infant head at the 5 and 8‐month time points for each Cohort. The triangles denote the primary ROIs identified during the Cluster Permutation Analyses to identify the region of maximal activation during the *Fam1* epoch. These ROIs substantially overlap one another with the UK cohort ROIs denoted in orange (light orange—5 months; dark orange—8 months) and the Gambia cohort ROIs denoted in green (light green—5 months; dark green—8 months)

Infants wore custom‐built fNIRS headgear consisting of two arrays (Figure 2), one over each of the left and right hemispheres (Lloyd‐Fox, Blasi, & Elwell, 2010). The arrays contained a total of 17 channels (source‐detector separations 2 cm) per hemisphere, and data were acquired with the NTS optical topography system (Gowerlabs Ltd. London, UK), which used source wavelengths of 780 and 850 nm. For the source detector separations used in this study and across this age range light transport models predict light penetration depths of up to approximately 1 cm from the skin surface, potentially allowing measurement of both the gyri and parts of the sulci near the surface of the cortex (Fukui, Ajichi, & Okada, 2003; Richards, Stevens, & Connington, 2012; Salamon, Raynaud, Regis, & Rumeau, 1990). Photographs of the participants were taken after the headbands were placed and at the end of the session to ensure proper placement and to facilitate offline coding of headgear placement and identification of measurement location relative to external scalp landmarks. Head measurements (head circumference, ear‐to‐ear measurements over forehead and over the top of the head) were also taken. With this information, and with the use of co‐registration MRI‐fNIRS data (Lloyd‐Fox et al., 2014) the underlying cortical anatomy of the fNIRS channels could be approximated in this study. The fNIRS arrays were designed so that responses could be recorded over auditory associative brain regions including the inferior frontal gyrus (IFG), middle and superior temporal regions and extending to the temporo‐parietal junction across both cohorts (Lloyd‐Fox et al., 2014). Head measurements were taken in each infant to enable the headgear to be aligned with 10–20 coordinates (Lloyd‐Fox et al., 2014).

Once the fNIRS headgear was placed on their heads, the infants sat on their parent's lap and the parent was instructed to refrain from interacting with the infant during the stimuli presentation unless the infant became fussy or sought their attention. During stimulus presentation an experimenter sat in front of the infant and silently interacted with them. They blew bubbles until the infant became inattentive and if necessary then moved on to show them soft toys, silently moving them back and forth. This interaction was continuous throughout the session designed to maintain infant's attention towards one target and to reduce head movements to a minimum while consistent across both experimental and baseline trials.

Auditory stimuli were an 8 s long spoken sentences (UK: ‘“Hi baby! How are you? Are you having fun? Thank you for coming to see us today. We're very happy to see you’, Gambian: “Denano a be nyadii. I be kongtan‐rin? Abaraka bake ela naa kanan njibee bee, n kontanta bake le ke jeh”. Two versions of the stimulus were recorded in each language by native English or Mandinka male and female speakers. The sentences were recorded in stereo at a sampling rate of 48 Khz, then edited using Audacity software v2.2.1 where they were normalized with a peak amplitude of −1dB and converted to mono. Stimuli were presented as part of a larger fNIRS battery of tasks, using a MATLAB® custom‐written stimulus presentation framework, Task Engine (sites.google.com/site/taskenginedoc) and Psychtoolbox (Brainard, 1997; Kleiner et al., 2007; Pelli, 1997), on an Apple Macintosh computer connected to Logitech Z130 stereo speakers. The intensity of sound was adjusted to a measured ~60dB at the position of the infant's head (range across the task: 60.1 dB–61.4 dB) at both sites.

Each stimulus sound was preceded by a 10 s silent period; this served as the baseline for fNIRS analyses. The trials were presented in the following order: 15 repetitions of a female speaker; 5 repetitions of a male speaker; and 5 repetitions of the same female speaker. These trials were then clustered into the following: Trials 1–5 (Familiarization 1‐*Fam1*); Trials 6–10 (Familiarization 2‐*Fam2*); Trials 11–15 (Familiarization 3‐*Fam3*); Trials 16–20 (*Novelty*); and Trials 21–25 (*Post‐test*).

In addition to the fNIRS study, anthropometric measures were performed. Infant lengths and weights were measured by using a Harpenden Infantometer length board (Holtain Ltd) and electronic baby scale (model 336; Seca), to a precision of 0.1 cm and 0.01 kg respectively. Mid upper arm circumference (MUAC) was measured by using a paper measuring tape to a precision of 0.1 cm. Head circumference, as a proxy for brain size, was measured to the nearest 0.1 cm with a stretch‐proof measuring tape (model201; Seca) around the maximum circumference of the head (forehead to occiput).

### 2.3 Data processing and analysis

Within each optical array, the NIR light measured by the detectors will have travelled from the sources through the skin & connective tissue, skull and underlying brain tissue layers. The NIRS system measured the light attenuation from each source detector pair. These light attenuation measures were used to calculate changes in the concentration of oxy‐haemoglobin (HbO_2_) and deoxy‐haemoglobin (HHb) in µMol which were used as haemodynamic indicators of cortical neural activity (Obrig & Villringer, 2003). The procedure of analysis, using in‐house programmes developed in MATLAB®, followed a similar protocol to previous infant research (Cristia et al., 2013; Gervain et al., 2011; Lloyd‐Fox et al., 2010). Data pre‐processing included a channel pruning step with several pre‐defined thresholds. The first threshold established a minimum DC value of attenuation reading of 3e‐4. This threshold is based on previous experience using the NTS system, and will exclude channels where there is not enough light from the source reaching the corresponding detector (e.g. due to hair blocking either optode, or one of the optodes being unclipped from the array). The second threshold sets the maximum acceptable difference between the coefficients of variation in the attenuation readings for the two wavelengths per channel (set at 0.2), therefore this criteria will exclude channels where the noise characteristics per wavelength are significantly different. Finally, a power spectrum density analysis of the raw signal will discard channels with strong frequency components unrelated to the experiment. For each infant, the raw intensity data were inspected using automated quality control scripts that included the above criteria, and channels with poor signal readings, excess variability in the data measured with the coefficient of variation, large baseline drifts or interference from the Eye Tracker (detected with a frequency analysis of the data) were excluded for further analysis. Infants with less than 60% of valid channels (21 channels for later time points) were excluded. The data were then divided into blocks consisting of 4 s of the baseline (silent) trial prior to the onset of the experimental trial, the experimental trial, plus the following baseline trial. The attenuation data were then detrended with a linear fit between the average of the first and the average of the last 4 s of each block. Following pre‐processing, data were converted into changes in concentration (µMol) in HbO_2_ and HHb using the modified Beer Lambert law (Delpy et al., 1988) and assuming a differential pathlength factor for infants (5.13; based on (Duncan et al., 1995)). A second level of automatic artefact detection and rejection was then conducted on a trial by trial level (within each channel) to identify excessive movement artefacts (concentration changes that exceeded a predefined threshold of ±3.5 µMol during the baseline and ±5 µMol during the experimental trials) in the concentration data.

**Figure 3:**
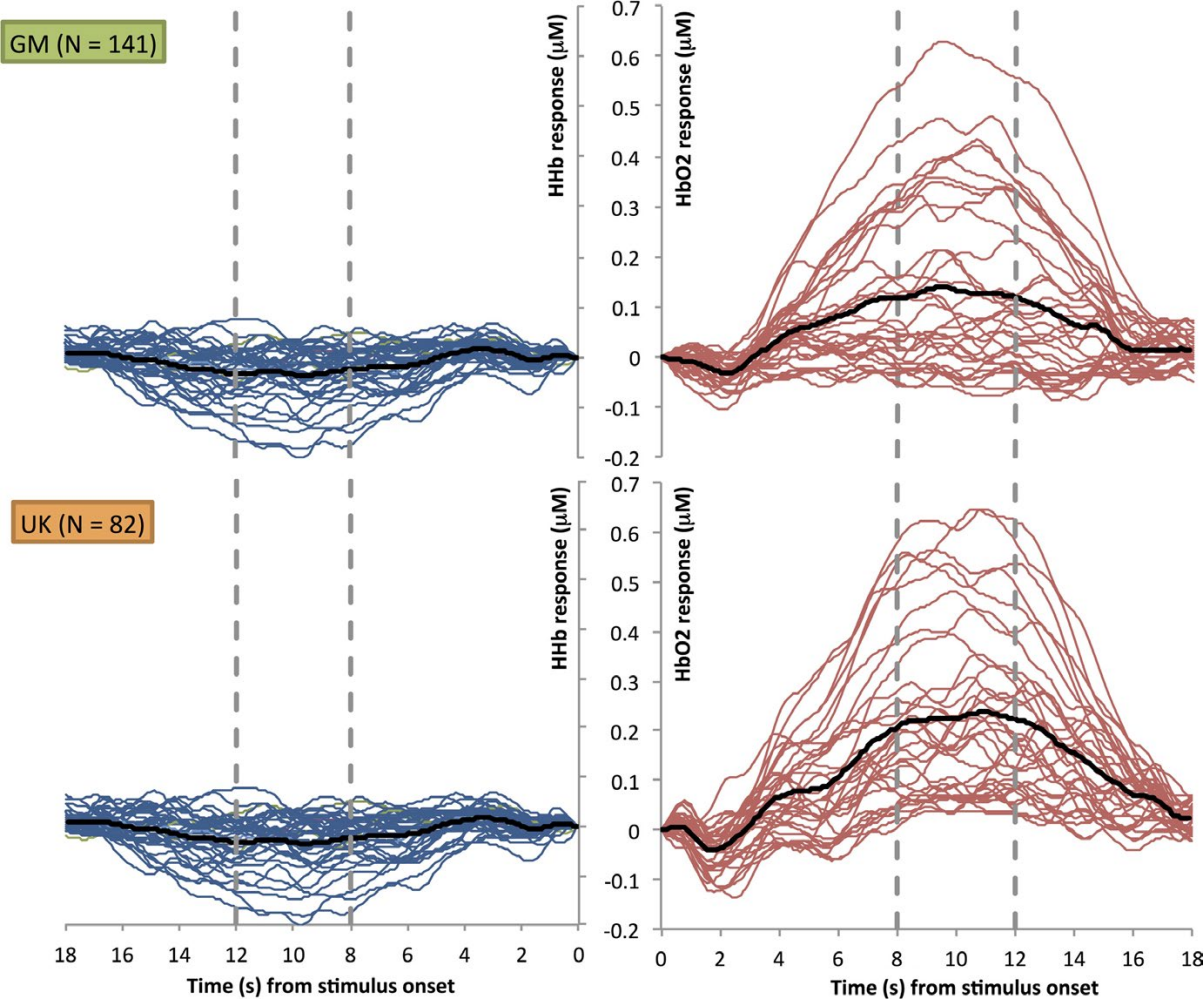
Grand averaged haemodynamic HbO_2_ (right) and HHb (left) time courses collapsed across age and epoch (*Fam1* and *Novelty*) and grouped per site; The Gambia (upper panel) and UK (lower panel). The black line indicates the grand average across all channels; the blue and red lines show the response per channel; and the dashed grey lines mark the start and end of the time window chosen for the statistical analyses

A minimum of three valid trials was set as a threshold for inclusion within each of the 5 epochs (i.e. *Fam1, Fam2, Fam3, Novelty, Post‐test*) per infant. The number of infants who were rejected at each age point, within each cohort, due to the channel and trial rejection criteria are described in Figure 1 (under the heading “motion artefact in data”). The trials and channels that survived these rejection criteria were then entered into further analyses. To be included in the first level channel by channel Habituation analyses, each infant had to have valid data in all three of the epochs (*Fam1, Fam2, Fam3)*within the Familiarization phase (therefore within an infant a minimum of 9 trials across the 15 trials were valid). For the Detection of Novelty analyses infants had to additionally have valid data in this phase. As a result additional infants were excluded from this secondary analysis: 2 at the 5 months time point and 1 at the 8 months time point, both for the Gambian cohort. The mean numbers of trials for each epoch (*Fam1, Fam2, Fam3, Novelty, Post‐test)* across ages were 5.00, 5.00, 5.00, 4.98, and 4.90 in Cohort 1 (UK) and 4.99, 5.00, 5.00, 4.87 and 4.73 in Cohort 2 (Gambia). For both analysis pathways valid trials within each epoch (*Fam1, Fam2, Fam3 and/or Novelty, Post‐test)* were averaged together within channels for each infant, and a time course of the mean concentration change in HbO_2_and HHb was compiled for each channel. A temporal window was selected between 8 and 12 s post‐stimulus onset for each epoch. This window was selected to include the range of maximum concentration changes observed across infants for HbO_2_ and HHb, based on visual inspection of the haemodynamic responses across all channels during *Fam1* and Novelty epochs (see Figure 3). Responses within this temporal window were compared to responses during an average of the final four seconds of the preceding baseline condition (i.e. inter trial interval of continuing visual experimenter interaction with no auditory stimulus). Either a significant increase in HbO_2_concentration, or a significant decrease in HHb, is commonly accepted as an indicator of cortical activation in infant work, however, in accordance with previous research (Lloyd‐Fox et al., 2010) the majority of the significant effects were in HbO_2_ and so results focused on this signal. Initially, for each channel, statistical comparisons (two‐tailed *t* tests) of the mean change (amplitude) within the 8 to 12 s time window (post stimulus onset) in HbO_2_(increase in chromophore concentration) were performed for all epochs (*Fam1, Fam2, Fam3, Novelty and Post‐test*) relative to the preceding baseline condition: these can be found in Supplementary Information. To resolve statistical problems of multiple comparisons for these group analyses the false discovery rate (FDR) correction (Benjamini & Hochberg, 1995) was applied.

To use a more data driven, yet anatomically informed approach, these t‐values were then entered into the main analyses using a Cluster based permutation approach (Maris & Oostenveld, 2007) to select the region of interest (i.e. the cluster of nearest‐neighbour channels with the strongest collective response to *Fam1*) for each Cohort at each age point. This approach provided a pathway to guide ROI selection, where no prior studies were available, and to confirm the *t* test results. Furthermore, the cluster‐based permutation analysis is a non‐parametric statistical test that provides a solution to the multiple comparisons problem for data collected simultaneously from multiple collection points close to each other in space (Maris & Oostenveld, 2007). This method has been previously used with fNIRS data to improve sensitivity to underlying activation (Abboub, Nazzi, & Gervain, 2016; Benavides‐Varela & Gervain, 2017; Ferry et al., 2016). Specifically to conduct the permutation analysis all possible sets of three triangulated nearest‐neighbouring channels (cluster candidates) along the fNIRS arrays (a total of 58 clusters) were identified. The cluster t‐value was then calculated by summing the *t* values (from the channel by channel *t*tests of the average response to *Fam1*relative to baseline) of each individual channel (spatial data point) within the cluster. The *t*values were selected from the analysis of the temporal window 8–12 s post stimulus onset: to test the temporal window chosen an analysis was also run on adjacent temporal windows at 4–8 and 12–16 s but found to be less informative. Following this the mean signal change was randomized by participant and channel, and new *t*‐values were calculated per channel and summed within each cluster to obtain the cluster *t‐*value. The data set was permuted (shuffled) randomly 1,000 times to generate a cluster probability curve of *t‐*values for the cluster. *N* = 1,000 permutations was chosen based on previous fNIRS research groups using this method (Abboub et al., 2016; Benavides‐Varela & Gervain, 2017). Clusters were selected that exceeded a threshold of *t* = 5 (see Supplementary data). The *t‐*value of each cluster candidate was then tested to see whether it was significantly different from chance using the random permutations of the data set: the cluster *p*‐value was then calculated as the area under the probability distribution to the right of the cluster *t*‐value. This was repeated for all candidate clusters. For each cohort, at each time point, the cluster within each array (left and right) with the most significant *p*‐value was selected. Given that the clusters identified in each hemisphere were over similar regions, and there were no a‐priori hypotheses about differential hemispheric habituation and novelty effects (as responses found in both hemispheres in previous research; Benavides‐Varela et al., 2011, Nakano et al., 2009), these were then combined across hemispheres to generate a primary bilateral ROI for the main analyses. Cluster‐based Permutation analyses were also conducted to investigate whether the location of the maximal response during the *Novelty* epoch relative to baseline differed to the location of that identified for initial responses during *Fam1*. Results of this analysis revealed very similar ROIs for both epochs. In all cases, the most significant cluster (ROI) always included channels placed over the temporal cortex. Therefore given the significant overlap all analyses focused on the ROI identified by the *Fam1* permutation analyses.

Within the primary ROI cluster for each cohort at each age point secondary analyses were performed to investigate (a) the presence of a habituation effect in the response, as indicated by a significant reduction in the amplitude of the signal change across *Fam1* to *Fam3*; and (b) the presence of a *Novelty Detection* response, as indicated by a significant recovery and subsequent increase during the *Novelty* phase relative to *Fam3*, but not the subsequent *Post‐test* phase. A repeated measures ANOVA was conducted with the factor *Epoch* (*Fam1*, *Fam3*, *Novelty* and *Post‐Test*) for each *Age point* (*5mo* and *8mo*) and each *Cohort* (UK and Gambia). Follow up pairwise comparisons were conducted between epochs per visit for any significant main effects found. Finally, a post hoc omnibus linear mixed model was also performed to assess the factors *Age at Visit* and *Hemisphere* for each Cohort.

## 3 RESULTS

Participant characteristics for age, gender and growth measures for the two Cohorts (UK and The Gambia) are presented in Table 1.

For the UK (Cohort 1) and Gambian (Cohort 2) cohorts firstly channel by channel *t*tests (FDR corrected) were run to investigate significant increases in HbO_2_ in response to the presentation of the auditory stimuli within each epoch (*Fam1, Fam2, Fam3, Novelty and Post‐test*) relative to baseline (silence). These data are presented in Supplementary Data.

T‐values from the *Fam1* versus baseline contrast were then used to conduct Cluster‐based Permutation Analyses to identify candidate 3‐channel clusters with *p*‐values <0.05 (see Methods). For the UK cohort 12 overlapping 3‐channel clusters were identified at the 5 month time point and 17 overlapping 3‐channel clusters at the 8 month time point. For the Gambia cohort 14 overlapping 3‐channel clusters were identified at the 5 month time point and 16 overlapping 3‐channel clusters at the 8 month time point (see Supplementary data). The permutation analyses revealed the primary ROI (comprised of the ROIs in each hemisphere with the most significant cluster‐level statistics, i.e. lowest *p*‐value) to be composed of a similar cluster of channels across both age points and across cohort (see Figure 2). In the UK at 5 months of age the primary ROI cluster covered channels 12, 13, 16, 31, 32 and 35 and at 8 months channels 12, 13, 14, 31, 32 and 35. In The Gambia at 5 months of age the ROI cluster covered channels 12, 13, 17, 31, 32 and 35 and at 8 months channels 12, 13, 16, 31, 35 and 36. These were located at an identical location bilaterally, positioned over the middle and superior temporal regions of the cortex.

**Figure 4:**
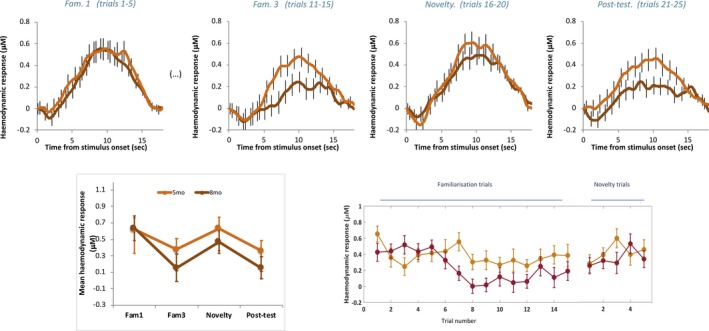
UK: Mean group haemodynamic HbO_2_ responses. Upper panel: mean group haemodynamic time courses for infants at 5 (orange) and 8 (red) months of age during *Fam1*, *Fam3*, *Novelty* and *Post‐test* epochs averaged across ROI channels. Lower panel: mean group haemodynamic HbO_2_ responses averaged across ROI channels during 8–12 s post stimulus onset for *Fam1*, *Fam3*, *Novelty* and *Post‐test* epochs (left panel) and for each presented trial (right panel) for infants at 5 (orange) and 8 (red) months of age. Error bars indicate ±*SEM*

**Figure 5:**
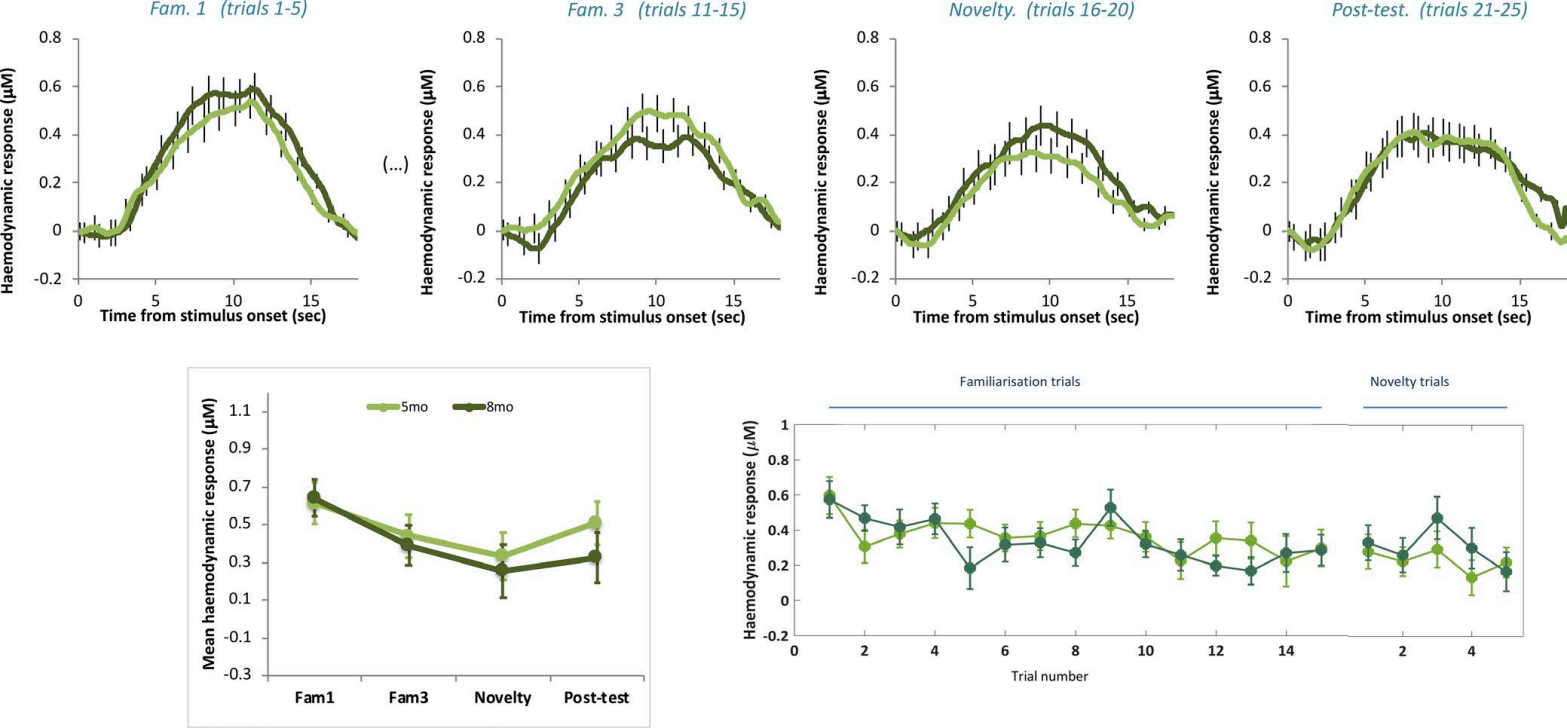
The Gambia: Mean group haemodynamic HbO_2_ HbO_2_ responses. Upper panel: mean group haemodynamic time courses for infants at 5 (light green) and 8 (dark green) months of age during *Fam1*, *Fam3*, *Novelty* and *Post‐test* epochs averaged across ROI channels (upper panel). Lower panel: mean group haemodynamic HbO_2_ responses averaged across ROI channels during 8–12 s post stimulus onset for *Fam1*, *Fam3*, *Novelty* and *Post‐test* epochs (left panel) and for each presented trial (right panel) for infants at 5 (light green) and 8 (dark green) months of age. Error bars indicate ± *SEM*

**Figure 6:**
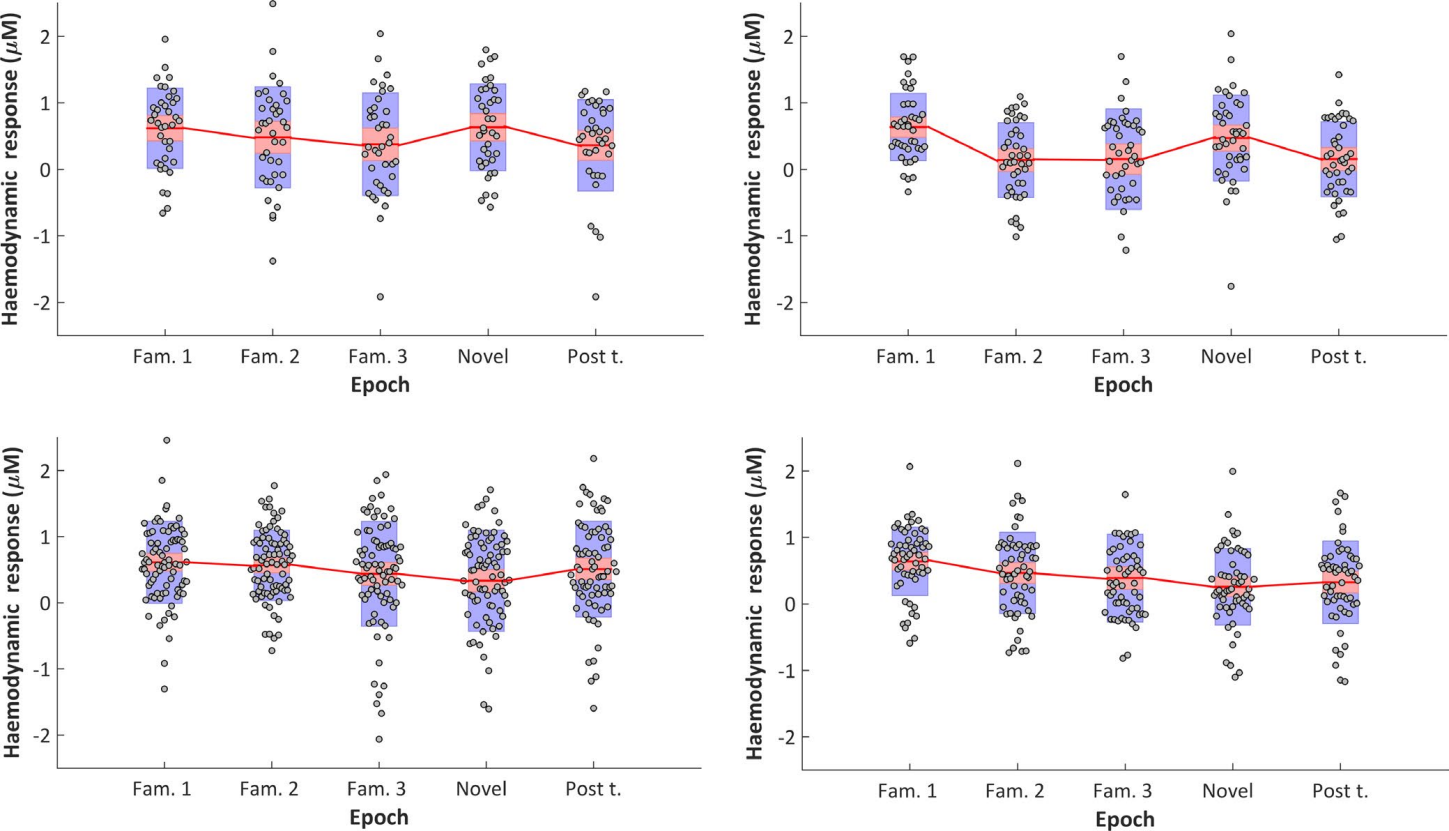
Mean individual haemodynamic HbO_2_ responses (±*SEM* (red box) and *SD* [blue box]) averaged across ROI channels during 8–12 s post stimulus onset, per condition epoch, for UK infants at 5 months (upper left panel) and 8 months (upper right panel) and for Gambian infants at 5 months (lower left panel) and 8 months (lower right panel)

Repeated measure ANOVAs within these ROIs for each cohort within each age group were conducted to investigate habituation and novelty detection responses (factor *Epoch* with four levels: *Fam1*, *Fam3*, *Novelty* and *Post‐test*). Finally, a post hoc omnibus linear mixed model was also performed to assess the factors *Age at Visit* and *Hemisphere* for each Cohort. The grand averaged responses within the time window of interest (8–12 s—see Figure 3) for each primary ROI across infants for the *Fam1, Fam3, Novelty and Post‐test*epochs, are shown in Figure 4 (UK cohort) and Figure 5 (Gambian cohort). Individual responses for each age point and cohort across all epochs are shown in Figure 6.

### 3.1 UK cohort

For the infant cohort in the UK at 5‐month of age, whilst responses are seen to follow a pattern of habituation and recovery to novelty at this age point (see Figure 4), overall the main effect of epoch was not significant (*F*(3) = 1.758, *p* = 0.168, η^2^ = 0.048; assuming sphericity: Mauchly's test *p* = 0.105). However, interestingly the pattern of habituation, with diminishing HbO_2_ responses from *Fam1* to *Fam3*, followed by response recovery (increase) to *Novelty* and a subsequent smaller response at *Post‐test*(see Figure 4), is indicated by a narrowly significant nonlinear (cubic) effect: *F*(3) = 4.127, *p* = 0.05, η^2^ = 0.105. Furthermore, the pairwise comparison between *Fam1* and *Fam3* is near significant (*t*(37) = 2.015, *p* = 0.051, *d* = 0.33). While these borderline significant results should be interpreted with caution, they are reported here as they follow the same pattern of significant responses seen at 8 months of age as described below.

By 8‐month of age the profile of the group response over epochs had become stronger and a main effect of *epoch* was found (*F*(3) = 7.618, *p* < 0.001, η^2^ = 0.157; assuming sphericity: Mauchly's test *p* = 0.396). Furthermore, the nonlinear (cubic) effect is significant (*F*(1) = 12.213, *p* = 0.001, η^2^ = 0.23). Pairwise comparisons revealed an effect of habituation evidenced by a significantly stronger response during initial familiarization trials in the *Fam1* epoch compared with *Fam3* (*t*(41) = 3.81, *p* < 0.0001, *d* = 0.76) and *Post‐test* (*t*(41)=4.304, *p* < 0.0001, *d* = 0.91) epochs. Furthermore, a recovery of response to novelty was evidenced by a significantly stronger response during the *Novelty* epoch relative to *Fam3* (*t*(41) = 2.526, *p* = 0.015, *d* = 0.46) and *Post‐test* (*t*(41) = 2.38, *p* = 0.022, *d* = 0.53) epochs.

Finally, an omnibus linear mixed model**—**with model dimensions Epoch (*Fam*1, *Fam3 and Novelty*), Hemisphere (*left and right*) and Age at Visit (*5mo or 8mo*) was conducted. This revealed a significant main effect of Epoch (*F*(38.23) = 13.11, *p* < 0.001). To explore the significant main effect further Bonferroni‐corrected post hoc pairwise comparisons were conducted, and revealed a significantly greater response to *Fam1* relative to *Fam3* (*p* < 0.001—*Fam1*: Mean = 0.619, *SEM* = 0.059 µMol; *Fam3*: Mean = 0.255, *SEM* = 0.078 µMol) and a significantly greater response to *Novelty* relative to *Fam3* (*p* = 0.02—*Novelty*: Mean = 0.571, *SEM* = 0.079 µMol).

### 3.2 Gambian cohort

For the infant cohort in The Gambia at 5‐month of age the profile of the response indicates continued habituation across epochs, evidenced by a main effect of epoch (*F*(3) = 2.717, *p* = 0.046, η^2^ = 0.035; assuming sphericity: Mauchly's test *p* = 0.7). In this case a nonlinear effect of epoch was also observed, although, in contrast to the UK cohort at 5‐months, it is quadratic instead of cubic (*F*(1) = 7.445, *p* = 0.008, η^2^ = 0.091) indicating a diminishing response with repetition of the stimulus trials across epochs. Pairwise comparisons revealed an effect of habituation which continued across Novelty trials, evidenced by a significantly stronger response during initial familiarization trials in the *Fam1* epoch compared with the *Novelty* epoch (*t*(77) = 2.849), *p* = 0.006, *d* = 0.41)). There are no other significant condition differences, therefore the habituation response was not evident during the initial *Familiarization* phase nor was there a recovery of response to *Novelty.*

For the infant cohort in The Gambia at 8‐months, similarly to at 5 months of age, the profile of the response indicates continued habituation across epochs (*F*(3) = 4.8, *p* = 0.03, η^2^ = 0.082, assuming sphericity: Mauchly's test *p* = 0.06). Pairwise comparisons revealed an effect of habituation which continued across epochs, evidenced by a significantly stronger response during initial familiarization trials in the *Fam1* epoch compared with the *Novelty* (*t*(55) = 4.25, *p* < 0.0001, *d* = 0.78) and *Post‐test* (*t*(53) = 3.775, *p* < 0.0001, *d* = 0.57) epochs. Similarly to at 5 months of age the habituation response was not evident during the initial Familiarization phase, nor was there a recovery of response to *Novelty.*

Finally, an omnibus linear mixed model**—**with model dimensions Epoch (*Fam*1, *Fam3 and Novelty*), Hemisphere (*left and right*) and Age at Visit (*5mo or 8mo*) was conducted. This revealed a significant main effect of *Epoch* (*F*(87.53) = 12.80, *p* < 0.0001) and a significant interaction of Epoch × Hemisphere × Age at Visit (*F*(62.12) = 4.58, *p* = 0.014). To explore the significant main effect further Bonferroni‐corrected post hoc pairwise comparisons were conducted, and revealed a significantly greater response to *Fam1* relative to *Fam3* (*p* = 0.001—*Fam1*: Mean = 0.654, *SEM* = 0.048 µMol; *Fam3*: Mean = 0.376, *SEM* = 0.056 µMol) and a significantly greater response of *Fam1*relative to *Novelty* (*p* < 0.001—*Novelty*: Mean = 0.318, *SEM* = 0.052 µMol). To explore the significant interaction further Bonferroni‐corrected post hoc pairwise comparisons were conducted per age point within each hemisphere, revealing differential patterns of activation. At *5‐months* of age the *left* hemisphere revealed a significantly greater response to *Fam1*relative to *Novelty* (*p* = 0.021—*Fam1*: Mean = 0.698, *SEM* = 0.095 µMol; *Novelty*: Mean = 0.293, *SEM* =  0.114 µMol), while no significant differences across Epochs were evident in the *right*hemisphere (*p* > 0.65). In contrast at 8‐month of age the *right* hemisphere revealed a significantly greater response to *Fam1*relative to *Fam3* (*p* < 0.0001—*Fam1*: Mean = 0.703, *SEM* = 0.086 µMol; *Fam3*: Mean = 0.323, *SEM* = 0.083 µMol) and relative to *Novelty* (*p* = 0.009—*Novelty*: Mean = 0.139, *SEM* = 0.10 µMol), whereas no significant differences across Epochs were evident in the *right*hemisphere (*p* > 0.18).

## 4 DISCUSSION

This study successfully implemented a novel habituation and novelty detection fNIRS infancy paradigm into a longitudinal study in The Gambia and the UK.

### 4.1 UK cohort

In accord with the hypotheses, in the UK cohort the analyses revealed three main findings. First, infants demonstrated a habituation (or repetition suppression) response to repeated auditory stimuli (spoken sentence), localized to middle and superior temporal regions of the cortex. Second, infants evidenced a novelty detection (or dishabituation) response to the presentation of the spoken sentence by a novel speaker in these same regions. Third, between 5 and 8 months habituation to repeated stimuli and recovery of response to novelty became increasingly evident, as the infants became older, indicative of developmental specialization.

In contrast to previous fNIRS research (Benavides‐Varela et al., 2011; Bouchon et al., 2015; Nakano et al., 2009)—where habituation and novelty detection responses were found in temporal and inferior frontal regions in infants 0–3 months of age—the main cluster permutation analyses and subsequent repeated measure ANOVAs did not demonstrate significant responses until 8 months of age. The predicted pattern of responses at five months of age was weaker with differential responses—indicative of habituation—evident at a borderline level during the ROI analyses and significant during the channel level preliminary analyses. The omnibus linear mixed model, however, did not find age‐related differences in activation patterns suggesting that overall the differences between the 5‐ and 8‐month‐old responses were not substantial. While the relative contribution to the current findings, of key differences between this study and previous research, cannot be fully teased apart, several factors can be considered in turn. First, and of import here, state of alertness (sleep vs. awake) may have significantly impacted the results. All previous research on habituation and novelty detection of brain responses using fNIRS has been conducted while infants sleep in contrast to this study which was undertaken while infants were awake. Interestingly, prior to their five and eight month visits infants also undertook the same study at 1 month of age while asleep. Preliminary analyses show that habituation responses were evident in this cohort at this earlier time point, similarly to previous research (Benavides‐Varela et al., 2011; Bouchon et al., 2015). Therefore the presentation of stimuli during sleep may alter the speed at which infants’ habituate to external stimuli (i.e. faster during sleep, no competing visual input interference). Second, it is likely that during the course of development the attention, learning and memory mechanisms associated with these responses will be impacted by the complexity and context of the study. While previous fNIRS studies have reported detection of novelty in the neonatal period (Benavides‐Varela et al., 2011; Bouchon et al., 2015; Mahmoudzadeh et al., 2013) here the response was found to be weaker at the younger age point (5 months relative to 8 months). The increased complexity in this study—including stimulus complexity; a change in the identity of a speaker during the presentation of a full sentence (compared to a novel word, speaker or tone), and environmental complexity; a naturalistic multimodal context with interfering external stimuli—may have therefore altered the age at which novelty responses in this context are seen. Third, different effects may have been found with a different arrangement of fNIRS channels across additional cortical regions—see Limitations section—though note that the fNIRS arrays used in this study cover the majority of prefrontal and temporal regions found to be selective regions in two previous fNIRS studies on novelty detection and habituation (Benavides‐Varela et al., 2011; Nakano et al., 2009).

Importantly, the overall study is designed to assess developmental trajectories across multiple longitudinal assessments during the first 2 years of life, rather than to establish whether infants can, or cannot, habituate/detect novelty to particular stimuli by a particular age per se. Therefore, as data continue to be collected across additional time points (12, 18 and 24 months), this will allow the investigation of developmental changes in repetition suppression and novelty detection across each cohort and the mapping of developmental trajectories within individual infants.

### 4.2 The Gambia cohort

At both 5 and 8 months of age, HbO_2_ responses evidenced habituation (repetition suppression) across the presented trials. Importantly, responses continued to decrease in amplitude across the *Familiarization* phase and the *Novelty* phase, with a significant habituation effect only evident between *Fam1* and *Novelty* epochs within the specific 5 and 8 month group analyses. Interestingly, when responses were combined across both age points in the omnibus modelling an overall main effect was found for Epoch, which when interrogated further did reveal significant decreases in the response by *Fam3* (relative to *Fam1*) as well as by the *Novelty* Epoch. Therefore at a cohort level, the habituation responses during the *Familiarization* phase were found to be similar across both the UK and Gambian infants in these post hoc contrasts. In contrast to the UK omnibus analysis, which showed a subsequent increase in response to the *Novelty* epoch, the Gambia omnibus modelling analysis revealed a continued decrease in response during the *Novelty* epoch. This indicates that at the group level infants did not perceive the change in stimuli as novel, as a recovery of response was not evident. Second, as shown by the post hoc analyses of the significant interaction found in the omnibus modelling analysis the habituation effects are complex. The repetition of 15 *Familiarization* trials was only sufficient to induce a habituation effect within the *right* hemisphere at 8 months of age. Given that this analysis revealed the strongest differential effects to be in the left hemisphere at 5 months of age, these lateralized findings should be treated with caution until further studies have been conducted, particularly given that previous fNIRS habituation and novelty detection studies have also not found consistent lateralization of effects (Benavides‐Varela et al., 2011; Nakano et al., 2009).

Overall, the findings from The Gambian cohort suggest less efficient habituation responses at 5 relative to 8 months of age (and relative to the UK) and an absence of a predicted novelty cortical response to a change in auditory stimuli. There are several possible explanations that should be explored. First, these findings could reflect less efficient specialization, or delayed development, of attention, learning and memory mechanisms (and thus a lower ability to filter irrelevant information and identify novelty), as has been found in EEG studies with older children living in low SES settings (D'Angiulli et al., 2008; Stevens et al., 2009). In previous work it was suggested that older children deploy supplementary resources to attend to irrelevant information (D'Angiulli et al., 2008) possibly reflecting a maturational lag in nervous system development or tonic cortical hypoactivation (Marshall, Fox, & Bucharest Early Intervention Project Core Group, 2004). Second, these findings could reflect adaptive responses within infants living in a very different environment relative to the environment of infants who partook in previously published findings. To take one example, infants may experience a higher/lower level of non‐directed speech in their environment, level of infant directed communication and number of primary caregivers. For example a recent study from The Gambia reported that within the villages in the West Kiang region family units can vary between 1 and 170 people, with an average of 16 members (Hennig et al., 2017), whereas in the UK an average household consists of 2.4 members (Office for National Statistics, 2016). Third, given that the environment of infants in rural Gambia may have led to an adaptive response which differs to that seen in the UK infants, it is possible that a different “neural solution” or network of regions relative to the areas currently identified are utilized for novelty detection (see Limitations section).

As additional longitudinal time points are measured in The Gambia, it will be important to investigate whether (a) these responses are developmentally delayed and become more typical by the next time points at 12, 18 or 24 months of age, (b) are adaptive to environmental factors and follow a different pathway, and (c) whether differential individual responses dependent on growth trajectories, or poverty associated risk factors, are driving the different pattern of longitudinal responses observed here. The vast majority of research of this kind undertaken to date has been within Western, Educated, Industrialised, Rich and Democratic (WEIRD) societies (Henrich et al., 2010). As this study continues in The Gambia, the increased sample sizes and age points in the Sub‐Saharan cohort will enable causal pathways of developmental specialization to be mapped in the presence of the diverse range of environmental and early adversity factors to which these infants are exposed. The collection of this data from as early in life as possible should allow the investigation of how different factors (such as maternal or infant undernutrition, family poverty) compound early development. Furthermore, the project aims to further understand how other factors (i.e. caregiving practices, day to day interactions) may compensate for the effects of, or allow adaptation to, these early risk factors. Therefore as this study continues the relationship between the development of repetition suppression and recovery of response to novelty and, to take one example, the complexity of the home environment (i.e. frequency of adult/child speech, environmental sounds and caregivers that the infant is exposed to) will be explored through the concurrent measurement of home caregiving interviews and assessments using automated language environment analysis (i.e. LENA). Furthermore, the project will draw together neurocognitive correlates of brain function from across different methodologies (EEG, attentional measures in eye‐tracking tasks, fNIRS) to further understand how trajectories of brain development observed within the habituation and novelty detection responses relate to other patterns of brain specialization across social, attention, memory, language and functional connectivity paradigms. This will allow us to further understand whether risk factors such as under nutrition differentially effect the development of emerging functional networks and regional hubs across the brain.

## 5 LIMITATIONS

Localization of responses: Previous research has found, in addition to the temporal region identified in this study, that right lateralized prefrontal areas were recruited during novelty detection (Benavides‐Varela, Hochmann, Macagno, Nespor, & Mehler, 2012; Nakano et al., 2009). Familiarization and novelty responses were not localized to prefrontal regions of the fNIRS arrays during analyses. However, in future work it could be important to extend coverage of the fNIRS arrays to explore novelty effects to a change in speaker further as the current arrays did not extend fully into dorsolateral areas of the frontal cortex. Importantly, this analytical approach allowed a data‐driven approach to isolate regions of interest. Therefore, in lieu of individual MRIs (which are not practically feasible in this context), this allowed the location of functional brain responses within the UK and The Gambia cohorts to be isolated to appropriate channel clusters separately. In future work, this could be independently run for different anatomical regions (i.e. temporal/frontal). Furthermore, as the relation between developmental trajectories of growth and brain function within The Gambian cohort are explored further the same approach can be used to identify ROIs at an individual level to accommodate differences in brain size and anatomical structure due to severe undernutrition.

Mapping of co‐variables: A limitation of the analysis presented in this paper is that it is currently limited to two time points (5 and 8 months of age) from a longitudinal study following children to 2 years of age. A primary objective of the BRIGHT study in The Gambia will be to assess the impact of longitudinal growth against brain development within this cohort. At this stage, there is not sufficient data to model growth across the period of key interest (birth to 2 years of age).

UK—Gambia comparisons: The aim of this study was to assess developmental specialization of brain function within cohorts, rather than draw direct comparisons between the UK and The Gambia. The UK cohort was included in this study to provide additional data regarding fNIRS longitudinal measures of the developmental specialization of the brain—in a more comprehensively researched population in the UK—so as to broadly match the context of previous research which has largely been conducted in “WEIRD” populations. As such the UK data set has allowed the successful measurement of age‐related changes in habituation and recovery to novelty brain responses in this new paradigm. In turn this will allow the investigation of the variability in responses within the Gambian cohort. Furthermore, through the continuation of data collection at 12, 18 and 24 months of age, individual trajectories of habituation and recovery of response to novelty will be stratified within The Gambian cohort across the first 2 years of life.

## 6 CONCLUSORY REMARKS

Importantly, neuroimaging measures such as fNIRS allows the identification of markers of atypical/adaptive function from a far earlier age (i.e. from birth) than behavioural assessments are typically able to (i.e. from 1 to 2 years onwards). The presented findings form part of a newly emerging field of research, which will in the future hopefully yield valuable insights into global neurocognitive development. Fundamentally by increasing understanding of longitudinal brain specialization in the UK, and developmental trajectories of brain function in The Gambia, this project will assess how infants’ react to their environment in ways allowing for an adaptive developmental outcome, thus resulting in a cortical structure and functioning most fit for their experienced circumstances (Johnson et al., 2015). The long‐term aim of this research is to establish fNIRS as a universal assessment tool for the investigation of the impact of adversity on cognitive development, identify individuals at greatest risk and target interventions from an early age before critical developmental milestones have been affected.

## ENDNOTE

1 The full sample has also now been recruited into the study in The Gambia: *N* = 223.
