# Supplementary material for: Habituation and novelty detection fNIRS brain responses in 5‐ and 8‐month‐old infants: The Gambia and UK
Source: Dev Sci. 2019 Mar 13;22(5):e12817. doi: 10.1111/desc.12817 (PMC6767511; doi:10.1111/desc.12817)
Supplement: Supplementary file 1 [file DESC-22-na-s001.docx]

SUPPLEMENTARY FIGURE 1: A schematic of the infant head showing statistically significant responses for the UK at 5 mo (left panel) and 8 mo (right panel) during the channel-by-channel analysis of the magnitude of change during the 12 – 16 second time window (t-test, two-tailed, p < 0.05, FDR corrected). The channels that revealed a significant HbO_2_ response in *Fam1* (yellow), *Fam2* (orange), and *Fam3* (red) are plotted in the upper panels. The channels that revealed a significant HbO_2_ response in *Novelty* (green) are plotted in the lower panels. Note: significant responses during the Novelty phase are only depicted when in the presence of concurrent diminishing response during the *Familiarisation* phase (i.e. no significant effects in *Fam3*) and without subsequent continued activation during the *Post-test* phase.


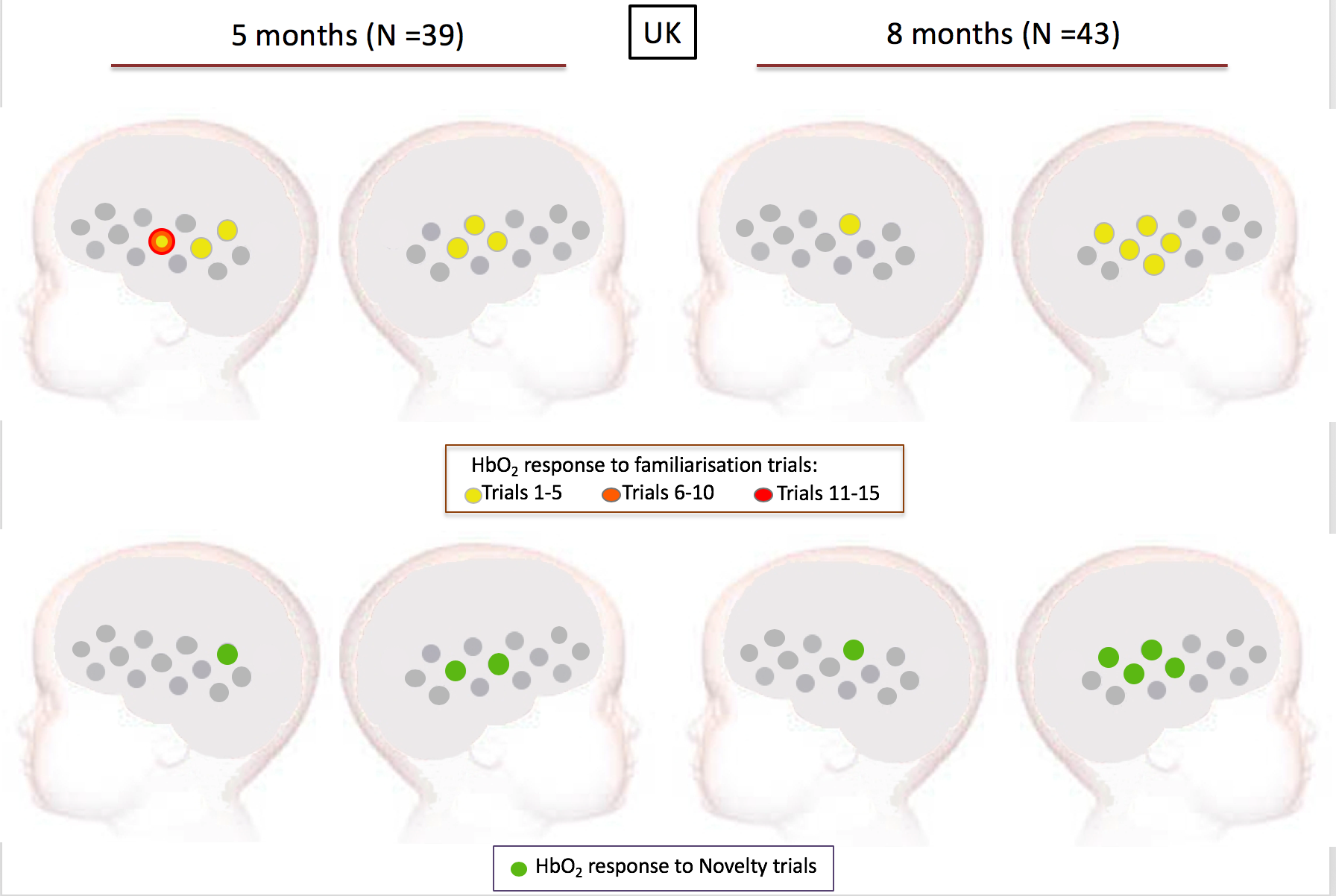


SUPPLEMENTARY FIGURE 2: A schematic of the infant head showing statistically significant responses for The Gambia at 5 mo (left panel) and 8 mo (right panel) during the channel-by-channel analysis of the magnitude of change during the 12 – 16 second time window (t-test, two-tailed, p < 0.05, FDR corrected). The channels that revealed a significant HbO_2_ response in *Fam1* (yellow), *Fam2* (orange), and *Fam3* (red) are plotted in the upper panels. The channels that revealed a significant HbO_2_ response in *Novelty* (green) are plotted in the lower panels. Note: significant responses during the Novelty phase are only depicted when in the presence of concurrent diminishing response during the *Familiarisation* phase (i.e. no significant effects in *Fam3*) and without subsequent continued activation during the *Post-test* phase.

SUPPLEMENTARY TABLE 1. Summary of HbO_2_ responses to Familiarisation (*Fam1, Fam2, Fam3*), *Novelty* and *Post-Test* Trials at 5 mo for UK and GM cohorts

SUPPLEMENTARY TABLE 2. Summary of HHb responses to Familiarisation (*Fam1, Fam2, Fam3*), *Novelty* and *Post-Test* Trials at 5 mo for UK and GM cohorts

SUPPLEMENTARY TABLE 3. Summary of HbO_2_ responses to Familiarisation (*Fam1, Fam2, Fam3*), *Novelty* and *Post-Test* Trials at 8 mo for UK and GM cohorts

SUPPLEMENTARY TABLE 4. Summary of HHb responses to Familiarisation (*Fam1, Fam2, Fam3*), *Novelty* and *Post-Test* Trials at 8 mo for UK and GM cohorts. There were no significant HHb responses for the UK cohort at 8mo

SUPPLEMENTARY TABLE 5. Candidate ROI clusters with p-values of less than 0.05 following the cluster permutation analyses for each cohort at 5 and 8 months of age. The primary ROI used for subsequent group analyses is shown at the top of the ranked table.

| **UK cohort (5 months)** | | | | | | **UK cohort (8 months)** | | | | |
| --- | --- | --- | --- | --- | --- | --- | --- | --- | --- | --- |
|  | ***Channels in cluster*** | | | ***t-value*** | ***p-value*** | ***Channels in cluster*** | | | ***t-value*** | ***p-value*** |
| ***Left hemisphere*** | ***12*** | ***13*** | ***16*** | **12.35** | **0.002** | ***12*** | ***13*** | ***14*** | **11.38** | **0.009** |
|  | *12* | *16* | *19* | 11.39 | 0.003 | *12* | *16* | *19* | 10.4 | 0.02 |
|  | *13* | *16* | *17* | 10.38 | 0.008 | *12* | *13* | *16* | 10.71 | 0.017 |
|  | *12* | *13* | *17* | 9.68 | 0.023 | *12* | *16* | *17* | 8.89 | 0.047 |
|  | *12* | *13* | *14* | 9.68 | 0.031 |  |  |  |  |  |
|  | *12* | *16* | *17* | 9.07 | 0.039 |  |  |  |  |  |
|  |  |  |  |  |  |  |  |  |  |  |
| ***Right hemisphere*** | ***31*** | ***32*** | ***35*** | **11.59** | **0.001** | ***31*** | ***32*** | ***35*** | **16.9** | **<0.001** |
|  | *31* | *35* | *38* | 10.48 | 0.012 | *31* | *35* | *38* | 15.3 | <0.001 |
|  | *31* | *32* | *33* | 9.48 | 0.035 | *32* | *35* | *36* | 13.97 | <0.001 |
|  | *31* | *35* | *36* | 8.48 | 0.039 | *31* | *35* | *36* | 14.35 | 0.001 |
|  | *32* | *35* | *36* | 8.80 | 0.039 | *31* | *32* | *33* | 13.57 | 0.002 |
|  | *34* | *35* | *38* | 8.4 | 0.044 | *34* | *35* | *38* | 11.45 | 0.006 |
|  |  |  |  |  |  | *31* | *36* | *38* | 12.0 | 0.006 |
|  |  |  |  |  |  | *29* | *32* | *33* | 10.38 | 0.015 |
|  |  |  |  |  |  | *34* | *35* | *36* | 10.5 | 0.018 |
|  |  |  |  |  |  | *35* | *37* | *38* | 9.37 | 0.029 |
|  |  |  |  |  |  | *28* | *32* | *33* | 9.45 | 0.03 |
|  |  |  |  |  |  | *28* | *29* | *32* | 9.21 | 0.043 |
|  |  |  |  |  |  | *28* | *32* | *36* | 9.47 | 0.046 |

| **Gambia cohort (5 months)** | | | | | | **Gambia cohort (8 months)** | | | | |
| --- | --- | --- | --- | --- | --- | --- | --- | --- | --- | --- |
|  | ***Channels in cluster*** | | | ***t-value*** | ***p-value*** | ***Channels in cluster*** | | | ***t-value*** | ***p-value*** |
| ***Left hemisphere*** | ***12*** | ***13*** | ***17*** | **14.94** | **0.004** | ***12*** | ***13*** | ***16*** | **16.08** | **0.003** |
|  | *12* | *13* | *16* | 16.01 | 0.005 | *12* | *13* | *17* | 13.82 | 0.008 |
|  | *13* | *16* | *17* | 14.70 | 0.005 | *12* | *16* | *19* | 12.5 | 0.016 |
|  | *12* | *16* | *17* | 14.72 | 0.008 | *12* | *16* | *17* | 12.82 | 0.02 |
|  | *12* | *16* | *19* | 13.42 | 0.023 | *13* | *16* | *17* | 11.94 | 0.033 |
|  | *9* | *13* | *17* | 13.08 | 0.025 | *12* | *13* | *14* | 11.55 | 0.037 |
|  |  |  |  |  |  |  |  |  |  |  |
| ***Right hemisphere*** | ***31*** | ***32*** | ***35*** | **17.39** | **<0.001** | ***31*** | ***35*** | ***36*** | **16.85** | **<0.001** |
|  | *28* | *32* | *36* | 16.07 | 0.003 | *28* | *32* | *36* | 15.09 | <0.001 |
|  | *32* | *35* | *36* | 16.39 | 0.003 | *32* | *35* | *36* | 16.51 | 0.001 |
|  | *31* | *32* | *33* | 13.06 | 0.015 | *31* | *32* | *35* | 12.31 | 0.002 |
|  | *28* | *32* | *33* | 12.70 | 0.016 | *34* | *35* | *36* | 14.02 | 0.003 |
|  | *31* | *35* | *36* | 13.30 | 0.022 | *31* | *35* | *38* | 13.18 | 0.018 |
|  | *28* | *29* | *32* | 12.77 | 0.025 | *31* | *32* | *33* | 12.95 | 0.018 |
|  | *31* | *35* | *38* | 11.3 | 0.038 | *28* | *29* | *32* | 12.43 | 0.019 |
|  |  |  |  |  |  | *28* | *32* | *33* | 11.93 | 0.025 |
|  |  |  |  |  |  | *31* | *36* | *38* | 11.98 | 0.028 |
